# Metnase Mediates Resistance to Topoisomerase II Inhibitors in Breast Cancer Cells

**DOI:** 10.1371/journal.pone.0005323

**Published:** 2009-04-24

**Authors:** Justin Wray, Elizabeth A. Williamson, Melanie Royce, Montaser Shaheen, Brian D. Beck, Suk-Hee Lee, Jac A. Nickoloff, Robert Hromas

**Affiliations:** 1 Division of Hematology-Oncology, Cancer Research and Treatment Center, Department of Medicine, University of New Mexico Health Science Center, Albuquerque, New Mexico, United States of America; 2 Department of Biochemistry and Molecular Biology, Indiana University School of Medicine, Indianapolis, Indiana, United States of America; 3 Department of Molecular Genetics and Microbiology, University of New Mexico School of Medicine, Albuquerque, New Mexico, United States of America; Health Canada, Canada

## Abstract

DNA replication produces tangled, or catenated, chromatids, that must be decatenated prior to mitosis or catastrophic genomic damage will occur. Topoisomerase IIα (Topo IIα) is the primary decatenating enzyme. Cells monitor catenation status and activate decatenation checkpoints when decatenation is incomplete, which occurs when Topo IIα is inhibited by chemotherapy agents such as the anthracyclines and epididophyllotoxins. We recently demonstrated that the DNA repair component Metnase (also called SETMAR) enhances Topo IIα-mediated decatenation, and hypothesized that Metnase could mediate resistance to Topo IIα inhibitors. Here we show that Metnase interacts with Topo IIα in breast cancer cells, and that reducing Metnase expression significantly increases metaphase decatenation checkpoint arrest. Repression of Metnase sensitizes breast cancer cells to Topo IIα inhibitors, and directly blocks the inhibitory effect of the anthracycline adriamycin on Topo IIα-mediated decatenation *in vitro*. Thus, Metnase may mediate resistance to Topo IIα inhibitors, and could be a biomarker for clinical sensitivity to anthracyclines. Metnase could also become an important target for combination chemotherapy with current Topo IIα inhibitors, specifically in anthracycline-resistant breast cancer.

## Introduction

Topo IIα inhibitors such as anthracyclines or epididophyllotoxins are important agents in the treatment of human malignancy [1]–[3]. These agents cause DNA damage by two mechanisms, locking Topo IIα in a cleavage complex producing DNA double-strand breaks (DSBs), and inhibiting chromatid decatenation [3]. While the former mechanism is well understood, far less is known about the latter, yet it can be just as catastrophic to the cell. Failure of decatenation results in DSBs at anaphase, and to prevent this cells probably monitor decatenation at two positions in the cell cycle, at the G_2_/M boundary and at the metaphase to anaphase transition [4]–[10]. These decatentation checkpoints are activated independently of the G_2_/M DNA damage-dependent checkpoint [3], [9], [11], [12]. Interestingly, lung and bladder cancers proceed through the decatenation checkpoints even in the presence of high levels of Topo IIα inhibitors, and this was thought to be secondary to a failure of the cell cycle arrest machinery [13], [14].

We recently isolated and characterized a human protein with SET and transposase domains called Metnase [15]. Metnase promotes non-homologous end joining DNA repair [15]–[18], enhances plasmid and viral DNA integration [18], and cleaves but does not degrade supercoiled plasmid DNA [19]. We recently showed that Metnase interacts with Topo IIα and enhances its function in chromosomal decatenation [19]. Therefore, we hypothesized that Metnase may mediate the resistance of malignant cells to Topo IIα inhibitors, and chose to test this in breast cancer cells because anthracyclines are among the most important agents in the treatment of this disease [1], [20]–[22]. We report here that Metnase interacts with Topo IIα in breast cancer cells, promotes progression through metaphase in breast cancer cells treated with a Topo IIα inhibitor, sensitizes breast cancer cells to the anthracycline adriamycin and the epididophyllotoxin VP-16, and directly blocks Topo IIα inhibition by adriamycin *in vitro*. These data indicate that Metnase levels may be one reason why some breast cancer cells treated with Topo IIα inhibitors can progress through mitosis without catastrophe resulting in drug resistance.

## Results and Discussion

**Figure 1:**
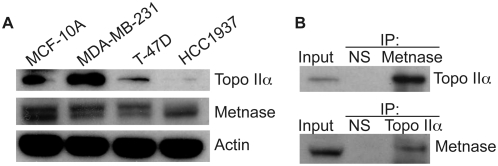
Topo IIα and Metnase interact in breast cancer cells. (A) MCF 10A, MDA-MB-231, T-47D, and HCC1937 were analyzed by Western blot for expression of Topo IIα and Metnase protein expression. Actin is shown as a loading control. (B) Metnase and Topo IIα were immunoprecipitated from MDA-MB-231 cells and analyzed by Western blot.

Previously, we showed that Metnase expression directly correlates with Topo IIα mediated decatenation in Human Embryonic Kidney cells. To determine if this finding would further apply to neoplasia, we evaluated Metnase and Topo IIα expression in four breast cell lines. MCF-10A is a cell line isolated from a benign hyperplastic breast lesion, T-47D from an infiltrating ductal carcinoma, HCC1937 from a primary ductal carcinoma, and MDA-MB-231 from a metastatic adenocarcinoma. As shown in Figure 1A, all of the cell lines express both Metnase and Topo IIα, though the HCC1937 have significantly reduced Topo IIα levels. Interestingly, MDA-MB-231 cells are the only cell line shown here derived from metastatic breast tissue. They have both an elevated Topo IIα level and significant Metnase expression. Because of this, we chose these cells to determine if Metnase and Topo IIα interact in breast cancer. In Figure 1B, we show that Metnase does co-immunoprecipitate (co-IP) with Topo IIα and that Topo IIα co-IPs with Metnase. Together, this provides evidence that Metnase could play a role in the pathogenesis and resistance of metastatic breast cancer to Topo IIα inhibiting therapies.

**Figure 2:**
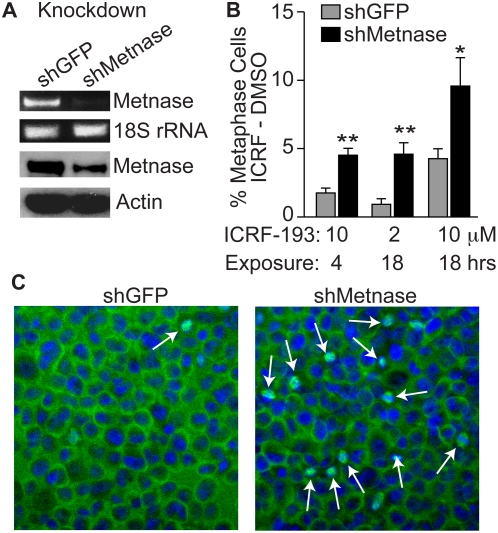
Metnase shRNA expression induces metaphase arrest in MDA-MB-231 cells. (A) Metnase expression was reduced by shRNA expression in MDA-MB-231 cells and analyzed by RT-PCR of mRNA and Western blot of protein, with 18S RNA and β-actin levels as loading controls. (B) Quantification of metaphase arrest after treatment with 2 or 10 µM ICRF-193 for 18 hrs. The percentages of metaphase cells in DMSO control cultures were subtracted from ICRF-193 treated cultures. Values are averages (+SEM) for 10–15 determinations; single asterisk indicates P = 0.022 and double asterisk indicates P = 0.0002 (t tests). (C) Representative images of control (shGFP) and Metnase knockdown cells expressing treated with ICRF-193 and stained with α-tubulin (green) and DAPI; arrows indicate metaphase cells.

Since Metnase enhances Topo IIα-mediated decatenation, and enhances resistance to ICRF-193 and VP-16 in non-malignant human cells [19], we hypothesized that Metnase might also promote resistance to the anthracyclines and epididophyllotoxins in MDA-MB-231 cells. We first investigated whether reducing Metnase would affect ICRF-193-mediated metaphase arrest. MDA-MB-231 cells were treated with ICRF-193, which inhibits Topo IIα after DNA religation, and therefore does not induce DSBs but does inhibit decatenation, allowing for discrimination between DNA damage and metaphase arrest [3]. The increase in cells arrested at metaphase in the presence of ICRF-193 compared to vehicle controls provides a measure of cells arrested due to failure of decatenation. Using α-tubulin immunofluorescence microscopy, we determined the fraction of cells in metaphase after exposure to ICRF-193. Cells with reduced Metnase expression (Fig. 2A) showed a significantly higher percentage of metaphase arrested cells when treated with ICRF-193 and cytospun onto slides to retain all cells (Fig. 2C). After 18 hour treatments with 2 or 10 µM ICRF-193, or 4 hours with 10 µM ICRF-193, cells with reduced Metnase showed 4.9-fold (p-value = 0.0016), 2.2-fold (p-value = 0.027), and 2.6-fold (p-value = 0.00015) increased metaphase arrest, respectively, as compared to vector control and evaluated by student's t-test (Fig. 2B). This result suggests that Metnase promotes decatenation in ICRF-193-treated MDA-MB-231 cells, allowing them to proceed through metaphase even in the presence of this Topo IIα specific inhibitor.

Prior studies revealed that bladder and lung cancer cells progress through the decatenation checkpoints when Topo IIα is inhibited by high concentrations of ICRF-193 [13], [14]. The conclusion from those studies was that these cancer cells failed to arrest because they had inactivated the decatenation checkpoints. While the ability to progress through mitosis even when Topo IIα is inhibited may be a general feature of malignancy, it may be due to the presence of Metnase alone, or Metnase in combination with checkpoint inactivation. Thus, the decatenation checkpoint may be intact in these malignant cells, but Metnase promotes continued Topo IIα function despite the presence of inhibitors, and the decatenation checkpoint is not activated.

**Figure 3:**
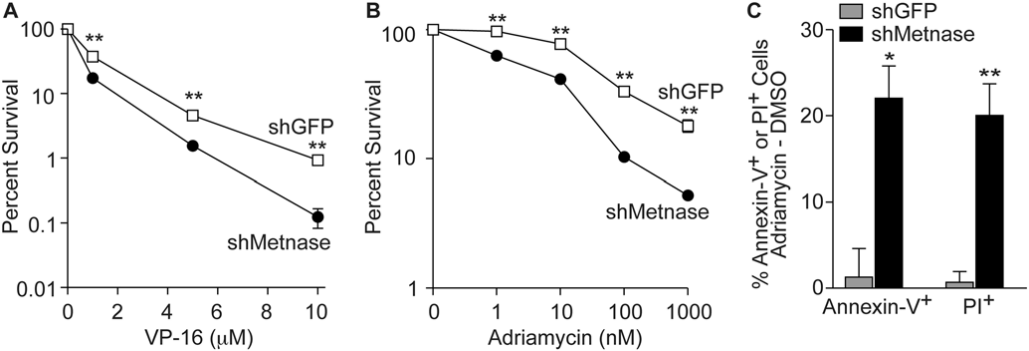
Metnase levels predict breast cancer cell sensitivity to Topo IIα inhibitors. Cells stably transfected with vector control, or Metnase shRNA were treated with VP-16 (A) or adriamycin (B) and colony survival was measured as described. Values are averages ±SEM for three determinations. (C) Cells transfected with control (shGFP) or Metnase shRNA were treated with 1 µM adriamycin for 24 hrs and apoptosis (annexin-V) and cell death (propidium iodide) were analyzed by FACS. Values are averages (+SEM) for three determinations.

The Topo IIα inhibitor ICRF-193 does not induce significant DNA damage, and therefore is not relevant in the clinical therapy of breast cancer. To determine whether altering Metnase levels would affect resistance to clinically relevant Topo IIα inhibitors, such as VP-16 and adriamycin [1], [2], we determined the cytotoxicity of these agents in MDA-MB-231 cell lines that stably under-expressed Metnase using colony formation assays. Decreased Metnase expression increased sensitivity 7.5-fold to VP-16, and 3.5-fold to adriamycin (Fig. 3A and B). Together, these results indicate that Metnase expression levels directly correlate with cell survival after exposure to these clinically relevant Topo IIα inhibitors. Adriamycin is an important agent in both adjuvant therapy and in the treatment of metastatic breast adenocarcinoma [1], so this finding is of relevance for current clinical regimens. It raises the possibility that treatment efficacy could be improved if the drug was used in combination with a future Metnase inhibitor, or if Metnase levels could be measured and possibly account for variance in responsiveness to adriamycin based chemotherapeutic regimens. Altogether, these results provide further support for the hypothesis that Metnase plays a key role in Topo IIα function.

To determine the mechanism for the ability of Metnase to mediate sensitivity to Topo IIa inhibitors, we investigated whether Metnase levels affected the cellular apoptotic response to adriamycin. We exposed MDA-MB-231 cells to adriamycin for 24 hrs and then evaluated annexin-V/FITC fluorescence by flow cytometry. We found that shRNA down-regulation of Metnase levels markedly sensitized these breast cancer cells to adriamycin-induced apoptosis (Fig. 3C). Compared to vector controls, cells with reduced Metnase levels showed a 17-fold higher frequency of apoptosis after adriamycin exposure. This finding suggests that Metnase suppresses adriamycin-induced apoptosis, contributing to the increased resistance of breast cancer cells to this drug.

**Figure 4:**
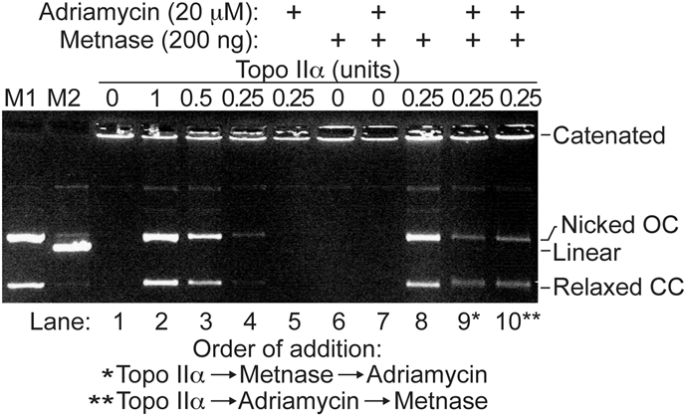
Metnase blocks the inhibitory effect of adriamycin on Topo IIα decatenation of kDNA. kDNA was incubated with varying amounts of Topo IIα (lanes 1–4), Topo IIα and adriamycin (lane 5), Metnase alone (lane 6), Metnase and adriamycin (lane 7), or Topo IIα and Metnase (lane 8). In lanes 9 and 10, kDNA was incubated with Topo IIα, Metnase and adriamycin with different orders of addition as indicated below.

To define the underlying mechanism of Metnase-dependent adriamycin resistance, we examined the effect of Metnase on adriamycin inhibition of Topo IIα-mediated decatention using a kinetoplast DNA (kDNA) *in vitro* decatenation assay (Fig. 4). Topo IIα decatenates kDNA (lanes 2–4) and adriamycin completely inhibits this activity (lane 5). As shown previously [19], purified Metnase does not decatenate kDNA on its own (lane 6), but enhances Topo IIα-dependent kDNA decatenation by 4-fold (lane 8). Importantly, when Metnase is present, it overcomes the inhibition of Topo IIα by adriamycin, and this is true whether Metnase is added to the reaction before or after adriamycin (lanes 9–10). Note also that in the presence of Metnase, there is a greater level of decatentation in the presence of adriamycin than with Topo IIα alone in the absence of adriamycin (compare lanes 9 and 10 with lane 4).

Metnase is a known component of the DSB repair pathway, and may enhance resistance to Topo IIα inhibitors by two mechanisms, enhancing DSB repair [15], [16] or enhancing Topo IIα function [19]. The data presented here suggest that the ability of Metnase to interact with Topo IIα, and enhance Topo IIα-dependent decatenation in vivo and in vitro may be at least as important as its ability to promote DSB repair in surviving exposure to clinical Topo IIα inhibitors. It is possible that Metnase could bind Topo IIα and physically block binding by adriamycin. In this model, Metnase would be bound to Topo IIα on DNA, and prevent adriamycin from stabilizing the Topo IIα/DNA cleavage complex, allowing Topo IIα to complete re-ligation. Alternatively, Metnase may function as a co-factor or chaperone to increase Topo IIα reaction kinetics. Here Metnase would bind transiently to Topo IIα and increase its reaction rate regardless of adriamycin binding. The mechanism may also be a functional combination of these two mechanisms where Metnase increases Topo IIα kinetics while also blocking further binding of the drug.

Our interpretation of these data is that Metnase increases the intrinsic function of Topo IIα via one of the above mentioned molecular mechanisms, and that this will result in fewer DSBs, not necessarily from enhanced DNA repair, but from Topo IIα directly resisting adriamycin inhibition and thus inhibiting the production of DSBs. This model is supported by our findings that Metnase significantly blocks breast cancer cell metaphase arrest induced by ICRF-193, and that cellular resistance to Topo IIα inhibitors is directly proportional to the Metnase expression level.

Our data reveal a novel mechanism for adriamycin resistance in breast cancer cells that may have important clinical implications. Metnase may be a critical biomarker for predicting tumor response to Topo IIα inhibitors. By monitoring Metnase levels, treatments with Topo IIα inhibitors may be tailored to improve efficacy. In addition, since reduced Metnase levels increase sensitivity to clinical Topo IIα inhibitors, inhibiting Metnase with a small molecule could improve response in combination therapies. Metnase inhibition may be especially important in a recurrent breast tumor that was previously exposed to Topo IIα inhibitors, since resistance to these agents may be due to upregulation of Metnase and/or Topo IIα. In summary, Metnase mediates the ability of Topo IIα to resist clinically relevant inhibitors, and may itself prove clinically useful in the treatment of breast cancer.

## Materials and Methods

### Cell culture, manipulating Metnase levels and co-immunoprecipitation

MDA-MB-231, T47, and HCC1937 breast cancer cell lines were cultured in Dulbecco's modified medium fully supplemented with 1% antimycotic/antibiotic (Cellgro, Mannasas, VA), and 10% Fetal Bovine Serum (Atlanta Biologicals, Lawrenceville, VA). The MCF10-A cell line was cultured in DMEM/F12 (Invitrogen, Carlsbad, CA) fully supplemented with 5% horse serum (Invitrogen, Carlsbad, CA), 20 ng/mL EGF (Invitrogen, Carlsbad, CA), 10 mg/L Insulin (Sigma, St. Louis, MO), 100 nM Hydrocortisone (Invitrogen, Carlsbad, CA), and 100 ng/mL Cholera toxin (Sigma, St. Louis, MO). MDA-MB-231 cells (ATCC) were stably transfected with pRS expressing shGFP control or shRNAs targeted to Metnase's nuclease domain in nucleotides 1198–1226, 1270–1298, and 1800–1828 (Origene, Rockville, MD). Metnase levels were assessed using RT-PCR and Western blotting [15]. Co-immunoprecipitation of Metnase and Topo IIα using anti-Topo IIα (Topogen, Port Orange, FL) was performed in the presence of 1.0 U/mL DNase I.

### Analysis of metaphase decatenation arrest

ICRF-193 (MP Biomedicals, Solon, OH) reversibly inhibits Topo IIα without inducing DNA DSBs, thereby allowing assessment of the decatenation checkpoints without activating the DNA damage checkpoints [7], [23]. ICRF-193 was dissolved in DMSO and further diluted in growth medium. Unsynchronized cells were treated with 2 or 10 µM ICRF-193, or an equivalent volume of DMSO (vehicle control) for 4 or 18 hours as labeled, harvested, cytospun, fixed with ice cold methanol, and stained with anti-tubulin antibody conjugated to FITC (Abcam, Cambridge, MA) for 2 hrs at room temperature and 4′-6-diamidino-2-phenylindole (DAPI, Vectashield). Interphase and metaphase cells were counted by immunofluorescence microscopy (Nikon TE2000 inverted microscope equipped with filter sets specific to FITC and DAPI). The fractional increase in ICRF-193 metaphase-arrested cells over vehicle controls was due to failure to traverse the metaphase decatenation checkpoint [3], [5], [8]–[10]. Data were collected from ≥1500 cells in duplicate experiments.

### Cytotoxicity of topoisomerase II inhibitors and analysis of apoptosis

Cells expressing various levels of Metnase were treated with VP-16 for 8 hr or adriamycin for 4 hr in growth medium, washed twice, fresh growth medium replaced, and incubated at 37°C with 5% CO_2_ for 10–14 days. Plating efficiencies were calculated by plating untreated cells in a similar manner. Colonies were stained with 1% methylene blue (Sigma, St. Louis, MO) and counted and percent survival was calculated after normalization to plating efficiency of untreated control cells. Apoptosis was analyzed by measuring annexin-V expression in cells treated with 1.0 µM adriamycin for 24 hr using the Annexin V-FITC Apoptosis Detection Kit I (BD Pharmingen, NJ).

### Kinetoplast DNA decatention

Purified recombinant Topo IIα (GE Healthcare, Piscataway, NJ) and catenated kinetoplast DNA (kDNA, Topogen, Port Orange, FL) were used according to the manufacturers' instructions. Recombinant Metnase was purified as described [24]. Adriamycin was added to specific reactions at a final concentration of 20 µM and kDNA decatenation was visualized by agarose gel electrophoresis.
